# Supplementary figures and images for: Expression of deubiquitinases in human gingiva and cultured human gingival fibroblasts
Source: BMC Oral Health. 2021 Jun 6;21:290. doi: 10.1186/s12903-021-01655-4 (PMC8180082; doi:10.1186/s12903-021-01655-4)

### Supplementary Figures

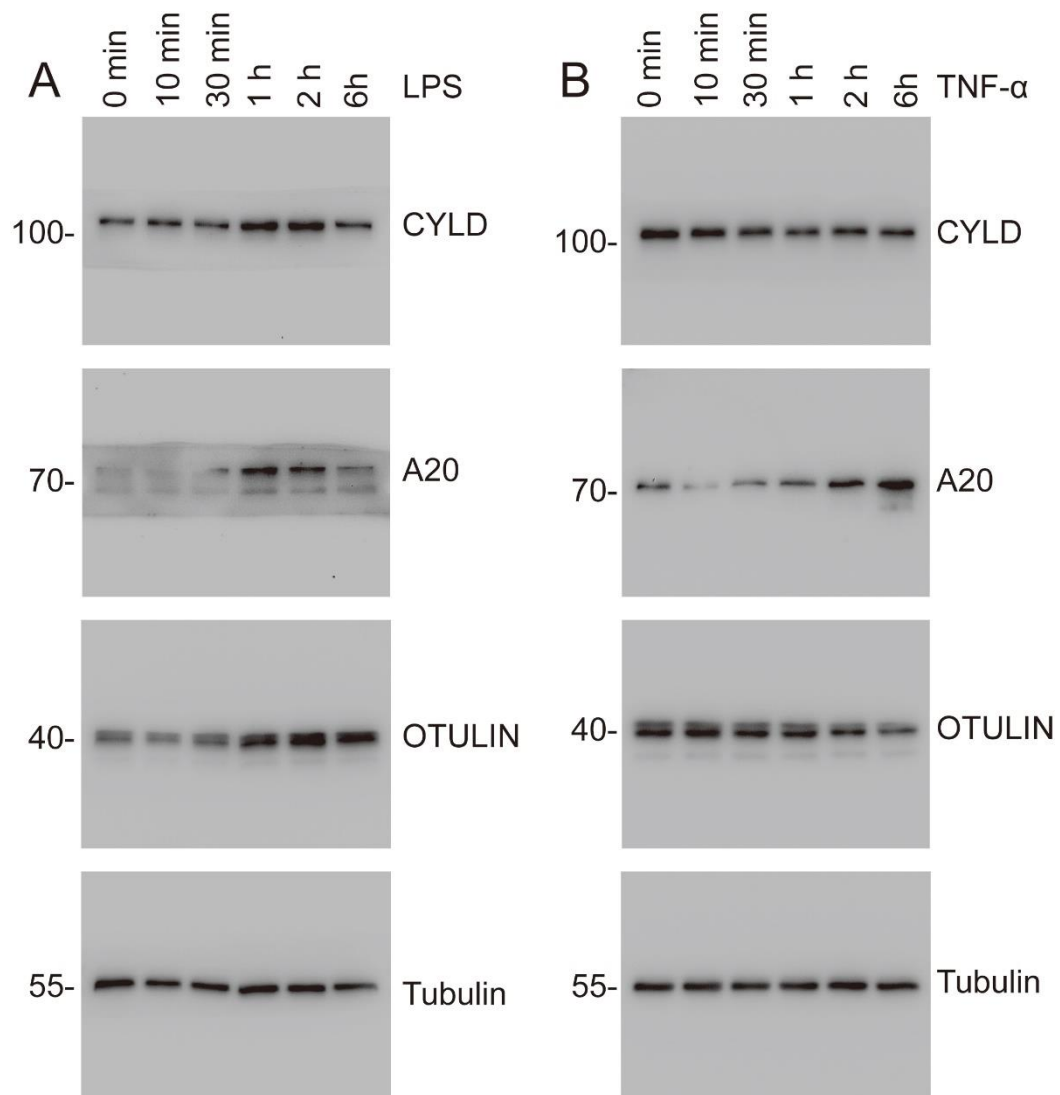

**Fig. S1** Full-length blots shown in Fig. 4

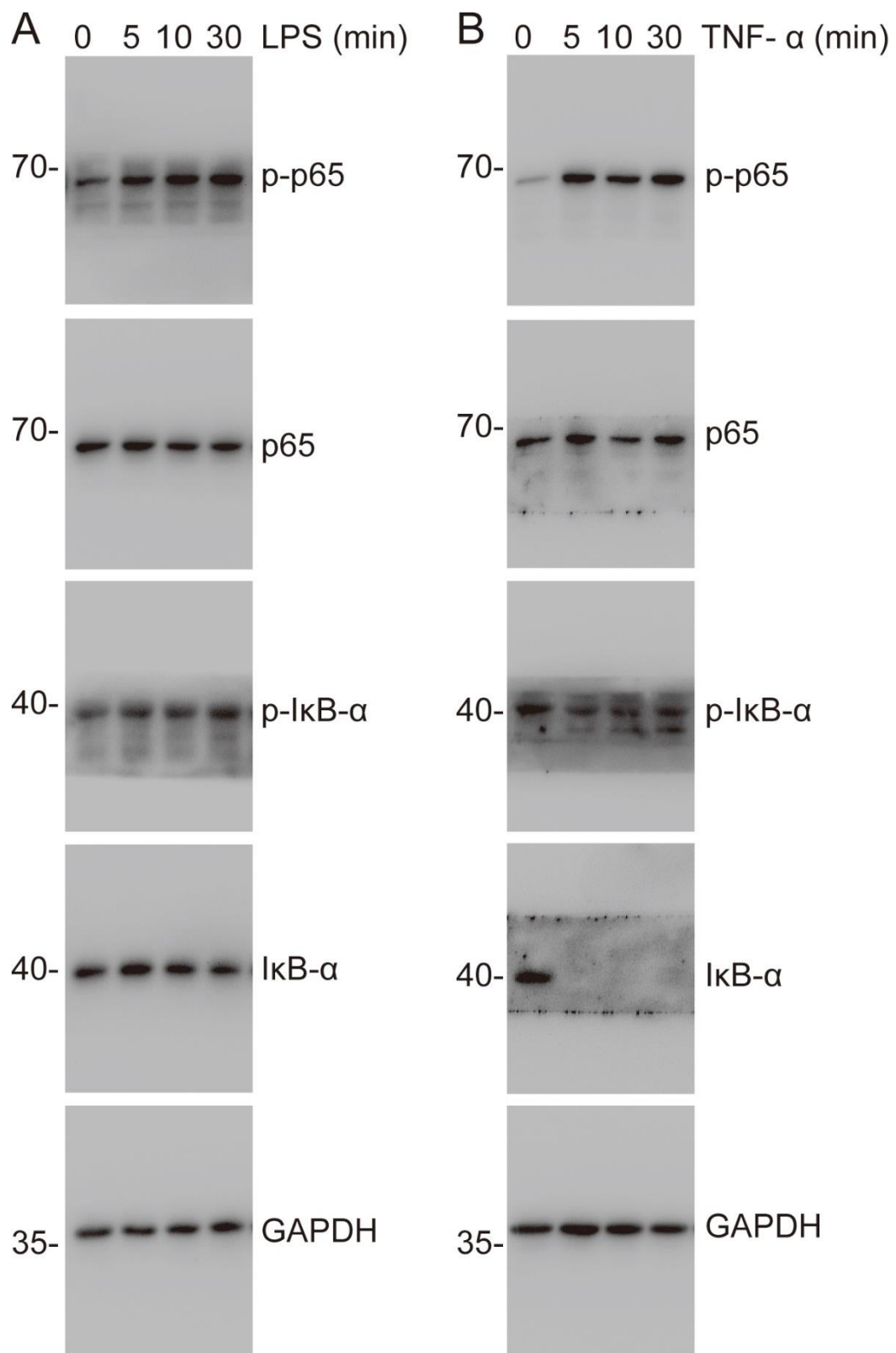

**Fig. S2** Full-length blots shown in Fig. 5

Supplement: Supplementary file 1 — Additional File 1. Full-length blots shown in Figs. 4 and 5. [file 12903_2021_1655_MOESM1_ESM.pdf]
